# On Spurious Diphtheria; Its Nature and Treatment

**Published:** 1864-10

**Authors:** 


### ON SPURIOUS DIPHTHERIA; ITS NATURE AND TREATMENT.

Much confusion is often caused, and many hindrances to the advancement of medicine are thrown in the way, by a loose and indiscriminate application of names; it ought, therefore, to be the aim of every one who has the interests of his profession truly at heart, to attain to the clear and distinct ideas as to the nature of disease, so that he may at all times think, and judge, and act, with precision. And one way in which he may assist in clearing up matters is, by studying diseases which are allied to each other, and by carefully observing and pointing out the distinguishing characteristics of each.

My object at present is to direct the attention of the profession to an affection which, in many respects, resembles diphtheria, and may be mistaken for it, but which, it will be found, differs essentially from that disease, both in its nature and its results.

During the prevalence of an epidemic, it is usually noticed that there is a strong tendency to a particular form of disease. When cholera prevails, for example, there are always many cases of severe diarrhœa and vomiting, which get well, and the true characteristics of the epidemic affection never become fully developed; in these there is a tendency to cholera, but it would be a misapplication of terms to call them real cholera cases, and so it is, I believe, in epidemics of diphtheria. Throat affections have a tendency to take on this form of disease, and many, many cases which are called diphtheria, and are even treated as diphtheria, are, I am convinced, merely examples of the affection I am about to describe.

How otherwise can we account for the apparent success of one practitioner in his treatment, and the total failure of another?

We hear of one man curing his diphtheria cases with one remedy, while another is equally successful with something totally different; but only let the boasted remedies be applied to a really serious case of true diphtheria, the diphtheria which Bretonneau studied so thoroughly, and has so graphically described, and I am convinced they will turn out to be utterly impotent and useless.

It is of importance then to distinguish between the two affections, and I shall now endeavor to sketch the characteristics of spurious diphtheria.

In the course of a recent outbreak of diphtheria, my attention was drawn to a certain class of cases which, while they presented some of the symptoms of that affection, never assumed such a serious nature, or called for such a vigorous plan of treatment, as did those which had previously come under my case.

In the class of cases to which I allude, the patient usually complains first of a curious feeling in the throat, as if a pin were pricking it; there is languor, with pains in the back and legs; and sometimes considerable tenderness on pressure on the outside of the throat, just under the angle of the jaw.

On looking at the throat, the tonsils and uvula are more or less tumefied, according to the severity or mildness of the case, and of angry red color, while on their surface small, irregularly shaped, yellowish white spots will be observed.

The spots are evidently of an aphthous nature; there may be only one or two on the tonsil or on the uvula, or they may be so numerous as to give to the soft palate an appearance as if some one had shaken a box of white pepper over it.

However great their number may be, I have observed that their edges do not coalesce, each spot is isolated. They never look excavated, but seem as if they just floated on the mucus which moistens the throat.

The appearance of the tongue usually indicates derangement of the digestive system, and the pulse is smaller and more frequent than in health.

The treatment of spurious diphtheria is exceedingly simple —a mild aperient, the tincture of the muriate of iron, in doses of ten or fifteen drops thrice a day, with a simple gargle of chlorine water, will certainly and speedily cure the throat affection. There may be a good deal of prostration and muscular debility after an attack of this disease, but a liberal diet and the use of stimulants, if necessary, will soon restore the patient to health.

*Aphorisms.*—Spurious diphtheria, so far as my observation has extended, never proves fatal. Though accompanied with debility, I have never seen it followed by paralysis or albuminuria; the tonsils sometimes suppurate after an attack. A patient who has suffered from this affection may subsequently be attacked with true diphtheria.

In true diphtheria gargles are of very little use till the patient begins to recover, but in the disease under consideration their use is always followed by the greatest benefit from the very first. In spurious diphtheria the use of caustic is not required. I do not know how to account for it, but this affection seems to be most prevalent amongst young females.—*Pacific Med. and Surg. Journal.*

